# The Role of Mitochondria in T-2 Toxin-Induced Human Chondrocytes Apoptosis

**DOI:** 10.1371/journal.pone.0108394

**Published:** 2014-09-29

**Authors:** Jiangtao Liu, Linlin Wang, Xiong Guo, Qingjiang Pang, Shixun Wu, Cuiyan Wu, Peng Xu, Yidong Bai

**Affiliations:** 1 School of Public Health, Science Health Center of Xi’an Jiaotong University, Key Laboratory of Environment and Genes Related Diseases, Ministry of Education, Xi’an, Shaanxi, PR China; 2 Department of Orthopedics Surgery, Ningbo No.2 Hospital, Ningbo, Zhejiang, PR China; 3 Department of Obstetrics and Gynecology, Ningbo First Hospital, Ningbo, Zhejiang, PR China; 4 Department of Orthopaedics Surgery, The Xi’an Red Cross Hospital, Xi’an, Shaanxi, PR China; 5 Department of Cellular and Structural Biology, University of Texas Health Sciences Center at San Antonio, San Antonio, Texas, United States of America; University of Sevilla, Spain

## Abstract

T-2 toxin, a mycotoxin produced by Fusarium species, has been shown to cause diverse toxic effects in animals and is also a possible pathogenic factor of Kashin–Beck disease (KBD). The role of mitochondria in KBD is recognized in our recent research. The aim of this study was to evaluate the role of mitochondria in T-2 toxin-induced human chondrocytes apoptosis to understand the pathogenesis of KBD. T-2 toxin decreased chondrocytes viabilities in concentration- and time-dependent manners. Exposure to T-2 toxin can reduce activities of mitochondrial complexes III, IV and V, ΔΨm and the cellular ATP, while intracellular ROS increased following treatment with T-2 toxin. Furthermore, mitochondrial cytochrome c release, caspase-9 and 3 activation and chondrocytes apoptosis were also obviously observed. Interestingly, Selenium (Se) can partly block T-2 toxin -induced mitochondria dysfunction, oxidative damage and chondrocytes apoptosis. These results suggest that the effect of T-2 toxin on human chondrocytes apoptosis may be mediated by a mitochondrial pathway, which is highly consistent with the chondrocytes changes in KBD.

## Introduction

T-2 toxin is a highly toxic trichothecene mycotoxin, and a naturally occurring mold byproduct of Fusarium species which is toxic to humans and animals [1]. At extremely high doses, tricothecenes can cause shock-like syndrome that can result in death. Dietary ingestion represents the most common route of human exposure. It is detected in a number of field crops (wheat, maize, barley and oats) and processed grains (malt, beer and bread) [2], [3]. Trichothecenes are now recognized as having multiple inhibitory effects on eukaryote cells, including inhibition of protein, DNA and RNA synthesis, inhibition of mitochondria electron transport system, mitochondrial function, and mitochondrial protein synthesis, effects on cell division and membrane effects [4]. In addition, T-2 toxin can decrease the level of seric antibodies, immunoglobulins as well as diverse cytokines [5]. Furthermore, T-2 toxin induced apoptosis has been considered to be one of the important mechanisms in its toxic effects [6].

Kashin–Beck disease (KBD) is a chronic, endemic osteochondropathy, which is mainly distributed in the area ranging from the northeastern to the southwestern China, as well as some regions in Russia and North Korea [7]–[9]. The disease is manifested as degradation of the matrix, cell necrosis mainly in the articular and growth plate cartilage, which can result in growth retardation, secondary osteoarthrosis, and disability in advanced stages [10], [11]. The T-2 toxin content remains at a high level in endemic grain and food, so serious cereal contamination by mycotoxin-producing fungi, especially T-2 toxin, was considerate as one of the most important probably etiology of KBD [12].

Mitochondria are membrane enclosed organelles found in most eukaryotic cells. Maintenance of the mitochondrial membrane potential (ΔΨm) and metabolizing enzyme activities is critical to adenosine triphosphate (ATP) synthesis [13], [14]. Depolarization of the ΔΨm increases the release of apoptotic factors from the mitochondria to the cytoplasm and leads to cell apoptosis [15]. Intracellular reactive oxygen species (ROS), one of several apoptotic factors, can augment oxidative stress and damage cells [16]. Bcl-2 is an anti-apoptotic protein [17]. A decrease in the ratio of Bcl-2 over Bax, an apoptotic protein, increases the risk that cells will undergo apoptosis [18].

Our previous study showed that T-2 toxin increases Bax protein production and induces chondrocyte apoptosis [19]. In addition, Our recent research demonstrated that articular chondrocytes from KBD showed a significant reduction in complex II, III, IV and V activities compared to normal chondrocytes, as well as decreased ΔΨm, but exhibited an increased mitochondrial mass [20]. What is the effect of T-2 toxin, as the probably etiology of KBD, to chondrocyte mitochondria is little known. Based on these results, we postulated that T-2 toxin could contribute to the mitochondrial alteration of chondrocytes and cartilage degradation, and would like mainly describe and preliminary evaluate the mitochondrial function in T-2 toxin-induced human chondrocytes apoptosis to understand the pathogenesis of KBD.

This study was designed to investigate the effect of T-2 toxin on the mitochondrial function, oxidative stress, and cytochrome c protein levels of human articular chondrocytes, and to analyze whether its effect on mitochondria is the mechanism by which it induces apoptosis. Further, we show that T-2 toxin induced apoptosis can be partially blocked through the addition of selenium.

## Materials and Methods

All studies were approved by the Institutional Review Board of Xi’an Jiaotong University. Informed-consent documents were written by the relatives of donors before entering this study.

### Cell culture and drug treatment

Specimens of human articular cartilage (age 45±12 years) were collected from a total of eight adult cadavers who had no history of joint disease and who had macroscopically normal cartilage. Slices of cartilage were aseptically dissected and chondrocytes were obtained by sequential digestions with hyaluronidase, crystallized trypsin and collagenase type II as previously described [20]. Cells were cultured to confluence at 37°C in a humidified atmosphere containing 5% CO_2_ in complete medium (DMEM with 15% fetal calf serum, 100 U/mL penicillin and 100 µg/mL streptomycin). All experiments were performed using the first passage chondrocytes to avoid any problems of dedifferentiation.

T-2 toxin purchased from Sigma Corporation (St. Louis, MO, USA) was freshly dissolved in DMSO and protected from light. Concentration-and time-dependent effects of T-2 toxin on chondrocytes were determined. The concentration of Se was chosen based on the normal range of serum levels (0.82–4.2 µM) of the population in China [21].

### Cell viability assay

Cells (1×10^4^) were seeded into individual 96-well plates and incubated under the above conditions. After 24 h of incubation, DMEM medium with or without 1–100 ng/ml T-2 toxin was added for different time periods (1–5 d). Then, 10 µL MTT at a final concentration of 500 µg/mL was added into the culture medium. After 4 h, the medium containing MTT was aspirated and replaced by the solution DMSO for 0.5 h. Following this incubation, the absorbance was measured in an ELISA reader at 550 nm. A preliminary study with concentrations of T-2 toxin up to 1000 ng/ml was performed, and the results determined that the appropriate concentrations for further studies were 0–100 ng/ml.

### Isolation of mitochondria

Mitochondria were isolated according to procedures previously described^20^. Chondrocytes (5×10^7^ cells) were collected by trypsinization, washed twice with PBS, and sedimented at 600 g for 5 min at 4°C. For mitochondria isolation, chondrocytes were incubated for 3 min in 10 mM Tris-HCl, pH 6.7, 10 mM KCl, and 1.5×10^−4^ M MgCl_2_ and were homogenized by 30 passes through a Potter-Elvehjem homogenizer. The homogenate was then resuspended in 0.25 M sucrose and centrifuged for 5 min at 1200× g to remove large debris and nuclei. The supernatants were centrifuged at 12,000× g for 15 min at 4°C, yielding a cytosol-enriched supernatant fraction and a mitochondrial pellet. The mitochondrial pellet was used to measure respiratory chain enzymatic activities and the cytosol fraction was reserved for the detection of cytochrome c.

### Evaluation of the citrate synthase (CS) activities and mitochondrial respiratory chain (MRC) complex activities

The measurement of specific respiratory chain enzymatic activities and CS levels were performed as described [22]. The rotenone-sensitive complex I activity was measured with 0.1 mM rotenone by following the decrease in absorbance due to the oxidation of reduced nicotinamide adenine dinucleotide (NADH) at 340 nm (ε = 6.22/mM/cm) in assay buffer by using a DU-640 spectrophotometer (Beckman Instruments, Palo Alto, CA, USA). Similarly, complex II was assessed by following the reduction of 2,6- dichlorophenolindophenol (DCPIP) at 600 nm (ε = 22/mM/cm). Complex III activity was measured by the reduction of cytochrome c at 550 nm (ε = 19.6/mM/cm). Complex IV activity was measured by the oxidation of reduced cytochrome c at 550 nm (ε = 19.6/mM/cm). Complex V was measured in the direction of ATP hydrolysis as oligomycin-sensitive ATPase (ε = 6.22/mM/cm). CS was evaluated at 412 nm (ε = 13.6/mM/cm) in 10 mM TriseHCl (pH 8), 10% Triton X-100, 50 mM oxaloacetic acid, 10 mM acetyl CoA, and 0.1 M 5,50-dithiobis(2-nitrobenzoic acid). The activities of the mitochondrial respiratory chain complexes were calculated as nmol/min/mg protein. The respiratory chain enzyme activity was normalized by dividing the enzymatic activity rates corresponding to each enzyme by the rate of CS activity.

### Quantification of the ΔΨm

ΔΨm was assessed using the fluorescent dye 5,5′,6,6′-tetrachloro-1,1′,3,3′- tetraethylbenzimidazole carbocyanide iodide (JC-1). Chondrocytes (1×10^6^) were collected by trypsinization, washed in PBS, and incubated for 15 min at 37°C with 20 µg/ml JC-1 (Molecular Probes, Eugene, OR, USA). Cells were pelleted at 600 g for 5 min, washed in PBS, and analyzed by flow cytometry using a FACScan and Cell-Quest software (Becton Dickinson, Mountain View, CA, USA). Photomultiplier settings were adjusted to detect JC-1 monomer fluorescence signals on the filter 1 (FL1) detector (green fluorescence, centered at ∼390 nm) and JC-1 aggregate fluorescence signals on the FL2 detector (red fluorescence, centered at ∼340 nm). JC-1 exists as a monomer at low values of ΔΨm (green fluorescence), while it forms aggregates at high ΔΨm (red fluorescence). Mitochondrial depolarization is indicated by a decrease in the red to green fluorescence ratio.

### Detection of cellular ATP contents

The contents of cellular ATP in chondrocytes were determined by a bioluminescence assay based on the luciferase requirement for ATP in producing emission light, according to the protocol for the Molecular Probes ATP Determination kit (Molecular Probes, Eugene, OR). 1×10^6^ cells were used in each analysis. The luminant light (560 nm) emitted by the luciferase-mediated reaction of ATP and luciferin was detected by a Wallac Victor 1420 multilabel counter (Welch Allyn, Turku, Finland).

### Determination of ROS

The production of ROS at mitochondrial and cytoplasmic level was assessed by MitoSOX red mitochondrial superoxide indicator and Carboxy-H_2_DCFDA probe (both from Invitrogen), respectively. In the 6-well plates, for each analysis 1×10^6^ cells were used. Briefly, after the addition of MitoSOX (2.5 mM) or Carboxy-H_2_DCFDA (20 mM), cells were incubated for 30 min at 37°C in the dark. Cells were washed and harvested in Hank’s buffered salt solution (HBSS) and analyzed immediately using a BD FACScan flow cytometer (Becton Dickinson, San Jose, CA) with excitation at 488 nm. Forward and side-scatter were used to gate the viable population of cells. Carboxy-H_2_DCFDA emit at 530 nm (FL-1) channel, whereas MitoSOX Red emit at 590 nm (FL-2 channel). A minimum of 10 000 events was collected. Data were analyzed as single parameter frequency histogram using cell Quest Alias software (Becton Dickinson, San Jose, CA). Results are presented as mean fluorescence intensity.

### Antioxidants activities analysis

The glutathione (GSH) content was determined with a GSH detection kit according to the manufacturer’s instructions (Jiancheng Bioengineering Institute, Nanjing, China). Briefly, the assay is based on the GSH recycling system by DTNB (5,50-dithiobis(2-nitrobenzoic acid)). DTNB and GSH react to generate 2-nitro-5-thiobenzoic acid which has yellow color. Therefore, the GSH concentration can be determined by measuring absorbance at a wavelength of 412 nm. Glutathione peroxidase (GPx) activity was measured according to an assay kit according to the instructions supplied by the manufacturer (Cayman Chemical, Ann Arbor, MI). Briefly, cellular extracts were measured for coupled oxidation of NADPH during GSH-mediated GSH-R recycling of oxidized GSH from GPX-mediated reduction of cumene hydroperoxide. The activity was normalized to protein content.

### Immunoblot analysis of cellular cytochrome c levels

Cytosolic proteins (50 µg per well) were subjected to 12% polyacrylamide gels, and electrophoretically blotted onto nitrocellulose membranes. Cytochrome c protein was immunodetected using a mouse monoclonal antibody against rat cytochrome c protein (Santa Cruz). β-actin was immunodetected by a mouse monoclonal antibody against mouse β-actin (Santa Cruz) as an internal control. Intensities of these immunoreactive protein bands were determined using a UVIDOCMW version 99.03 digital imaging system (UVtec, Cambridge, UK).

### Fluorogenic substrate assay for caspase-9 and 3 activities

Activities of caspase-9 and 3 were determined using fluorogenic assay kits (R&D Systems, Minneapolis, MN). Human chondrocytes were lysed, and cell extracts (25 µg total protein) were incubated with 50 µM specific fluorogenic peptide substrates, Leu-Glu-His-Asp-7- amino-4-trifluoromethyl coumarin (LEHD-AFC) and Asp-Glu-Val-Asp-7-amino-4- trifluoromethyl coumarin (DEVD-AFC) for caspase-9 and 3, respectively. Intensities of fluorescent products were measured by the LS 55 spectrometer of PerkinElmer Instruments (Shelton, CT, USA).

### Annexin V staining for chondrocyte apoptosis

Annexin V staining for chondrocyte apoptosis. Annexin V in combination with PI was used to distinguish apoptotic chondrocytes from necrotic chondrocytes in monolayer cultures. Following treatment, chondrocytes were labeled using the Annexin V-FITC Apoptosis Detection kit according to the manufacturers’ instructions (Becton Dickinson, CA, USA). For assessment of apoptosis, cells were resuspended in staining solution and incubated with annexin V and PI in the dark for 15 minutes at room temperature, and analyzed by FACScan. The apoptotic percentage of 10,000 cells was determined and all experiments reported in this study were performed in triplicate.

### Statistical analysis

Results are expressed as means±SD. Data were evaluated with one-way analysis of variance (ANOVA) procedures among treatment groups. Each experiment was done at least three times separately. Differences were considered significant at *P*<0.05.

## Results

### Effect of T-2 toxin on chondrocyte survival

**Figure 1:**
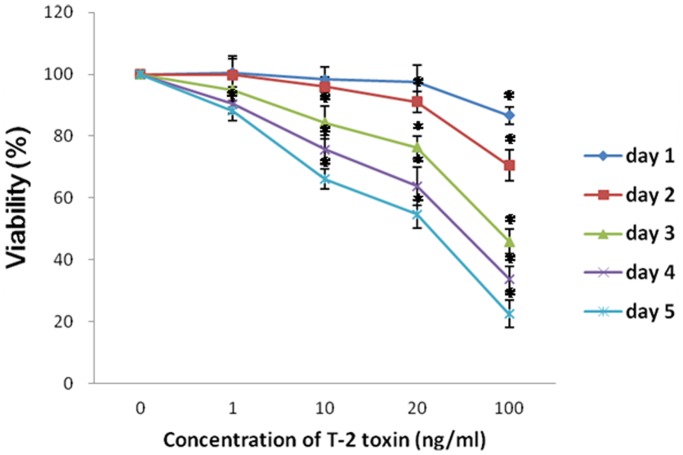
Effects of different concentrations of T-2 toxin on the cellular viability of chondrocytes were estimated by MTT reduction. Cells were incubated in absence or presence of several T-2 toxin concentrations for different time periods (1–5 d). *P<0.05.

Chondrocytes were exposed to increasing concentrations of T-2 toxin (1–20 ng/ml) for different time (1–5 days), and cell viability was determined by MTT assay (Fig. 1). T-2 toxin induced a time- and dose-dependent inhibition of cellular proliferation in chondrocytes. We chose the exposure time of 5 days in the subsequent experiment because all concentrations of T-2 toxin used (1–20 ng/ml) significantly decreased the cellular proliferation after 5 days of exposure. Based on the MTT assay, the IC50 value at 5 day exposure of T-2 toxin was about 20 ng/ml, all future experiments were accordingly carried out at 3 T-2 toxin concentrations (1, 10, 20 ng/ml) for a maximum for biochemical analysis.

### Effects of T-2 toxin on mitochondrial function

Mitochondrial function was evaluated by analyzing the enzymatic activity of respiratory chain complexes, changes in ΔΨm, intracellular ATP contents, as well as the level of intracellular ROS. In relation to the enzymatic activity of the MRC, T-2 toxin significantly reduced the activity of complex III, IV and V (Table 1) in normal human chondrocytes. However, T-2 toxin did not affect the activity of complexes I, II, or that of CS. Meantime, selenium could partly block the effect of complex IV and V activities induced by T-2 toxin.

**Table 1:** Values of mitochondrial respiratory chain complexes in cultures of normal chondrocytes treated with 20 ng/ml T-2 toxin for 5 days.

	Control	20 ng/ml T-2	20 ng/ml T-2+1 µM Se
n	8	8	8
Age (years old)	45.38±8.78	45.38±8.78	45.38±8.78
Proteins (mg/ml)	2.98±0.47	2.55±0.68	2.75±0.55
CS enzymatic activity (nmol/minute/mg protein)	95.39±15.62	88.99±12.21	94.21±13.70
Mitochondrial complex activity†			
Complex I	33.54±6.84	28.71±5.30	30.94±6.84
Complex II	13.65±2.58	11.24±1.94	12.64±2.53
Complex III	55.35±7.29	47.70±3.88*	53.32±5.24
Complex IV	50.41±5.79	39.91±5.36*	46.65±6.50#
Complex V	53.44±9.84	42.26±8.46*	51.95±8.83#

Values are the mean ± SD. CS = citrate synthase. †CS-corrected complex activity is expressed as (nmoles/minute/mg protein)/(CS specific activity) ×100. *P<0.05 versus normal chondrocytes. #*P*<0.05 versus 20 ng/ml T-2 toxin group. Complex I = rotenone-sensitive NADH-coenzyme Q1 reductase; complex II = succinate dehydrogenase; complex III = antimycin-sensitive ubiquinol cytochrome c reductase; complex IV = cytochrome c oxidase; complex V = ATP synthase.

**Figure 2:**
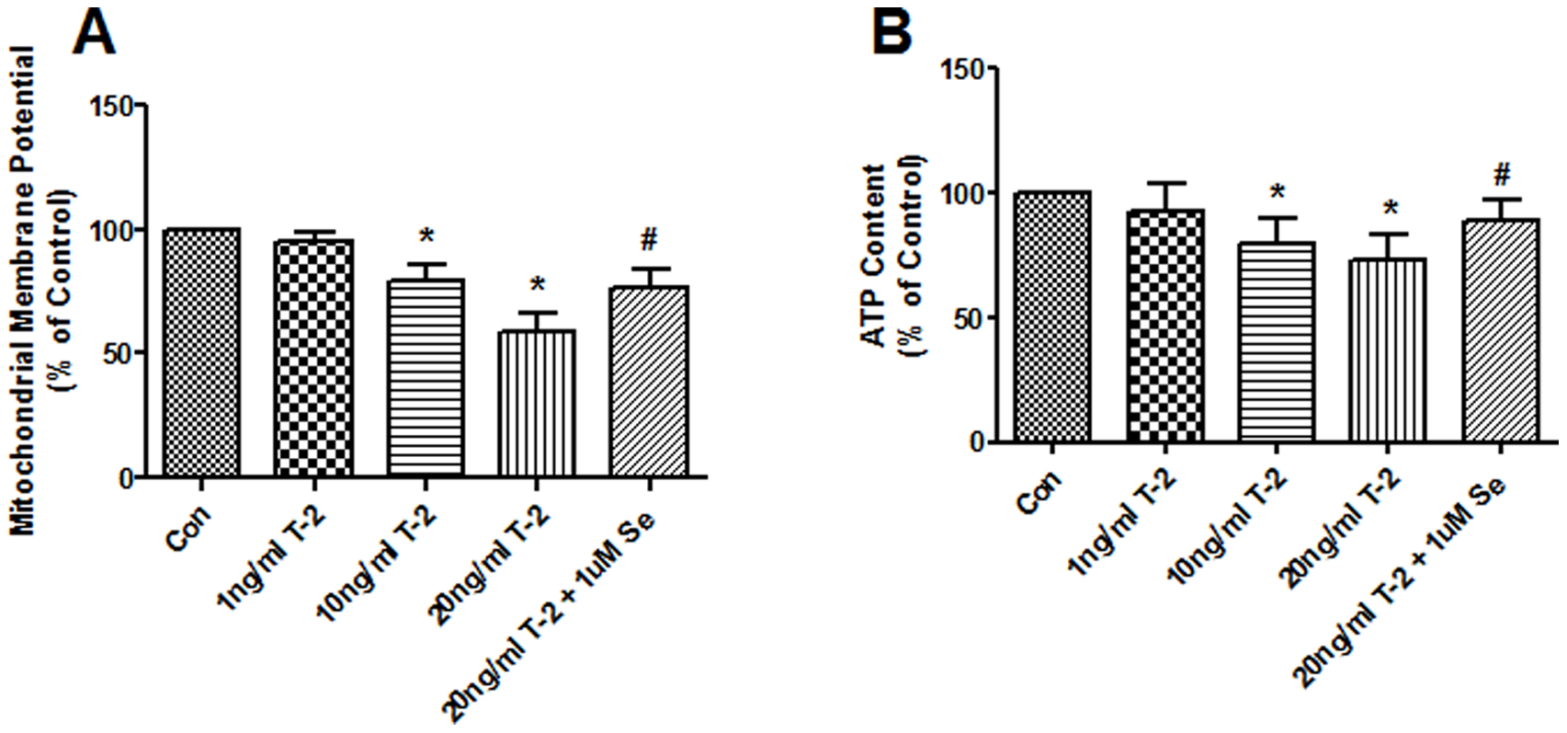
Effects of T-2 toxin on the mitochondrial membrane potential and ATP. **A.** Normal human chondrocytes were exposed to 1 10, 20 ng/ml T-2 toxin and 1 uM Na_2_SeO_3_+20 ng/ml T-2 toxin for 5 d. The mitochondrial membrane potential of chondrocytes was analyzed using the fluorescent dye JC-1, and quantified by a flow cytometer. **B.** Effect of different concentrations of T-2 toxin on the cellular ATP. *P<0.01 vs. control; #*P*<0.01 vs. 20 ng/ml T-2 toxin group.

The ΔΨm and cellular ATP levels of chondrocytes was determined and shown in Figure 2. In l ng/ml treated chondrocytes, T-2 toxin did not change the ΔΨm. After administration of T-2 toxin for 10 and 20 ng/ml, the ΔΨm was significantly decreased by 21% and 41%, respectively (Fig. 2A). Similar trends to the ΔΨm, l ng/ml T-2 toxin did not change the cellular ATP levels. After administration of T-2 toxin for 10 and 20 ng/ml, cellular ATP levels were significantly decreased by 20% and 27%, respectively (Fig. 2B). When cells were pretreated with 1 µM selenium for 30 min, the ΔΨm and ATP of chondrocytes increased by 30% and 16%, separately.

**Figure 3:**
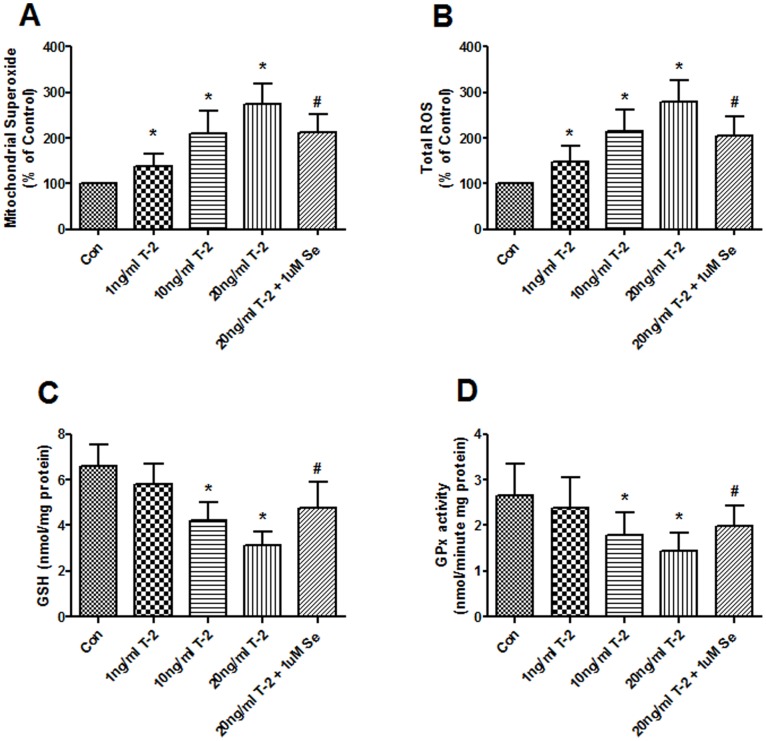
Effects of T-2 toxin on ROS and antioxidants generation. The production of ROS at mitochondrial (**A**) and cytoplasmic (**B**) level was assessed by MitoSOX red mitochondrial superoxide indicator and Carboxy-H_2_DCFDA probe (both from Invitrogen), respectively. GSH concentration (C) and GPx activity (D) were measured by coupled enzyme assay as described under “Materials and methods”. Chondrocytes were incubated with1, 10, 20 ng/ml T-2 toxin and 1 uM Na_2_SeO_3_+20 ng/ml T-2 toxin for 5 d. *P<0.01 vs. control; #*P*<0.01 vs. 20 ng/ml T-2 toxin group.

T-2 toxin significantly increased the ROS both at mitochondrial and cytoplasmic level in all of 1, 10 and 20 ng/ml groups after treatment chondrocytes for 5 days (Fig. 3A and B). However, the intracellular GSH concentration and GPx activity were significantly reduced in 10 and 20 ng/ml T-2 toxin groups (Fig. 3C and D). Pretreatment with 1 uM Na_2_SeO_3_ attenuated the increased ROS and blocked the decreased GSH and GPx.

### Effect of T-2 toxin on mitochondria-mediated apoptosis

**Figure 4:**
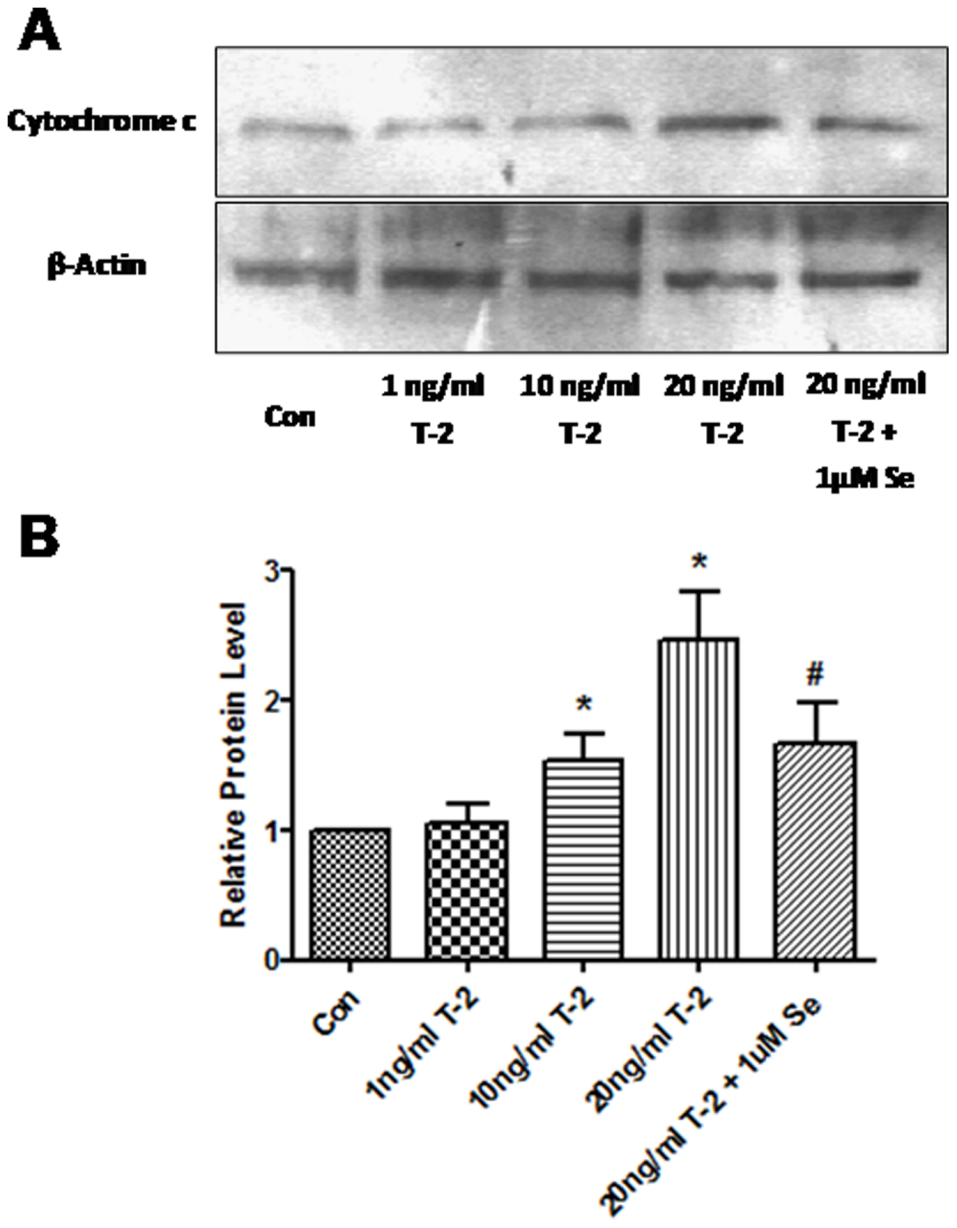
Western blot analysis for cytochrome c protein in normal human chondrocytes after treatment with 1, 10, 20 ng/ml T-2 toxin and 1 uM Na_2_SeO_3_+20 ng/ml T-2 toxin for 5 d. **A.** Western blot of cytochrome c. **B.** Increase of cytochrome c relative to control. Data are shown as the mean ± S.D. of at least three separate experiments. *P<0.01 vs. control; #*P*<0.01 vs. 20 ng/ml T-2 toxin group.

**Figure 5:**
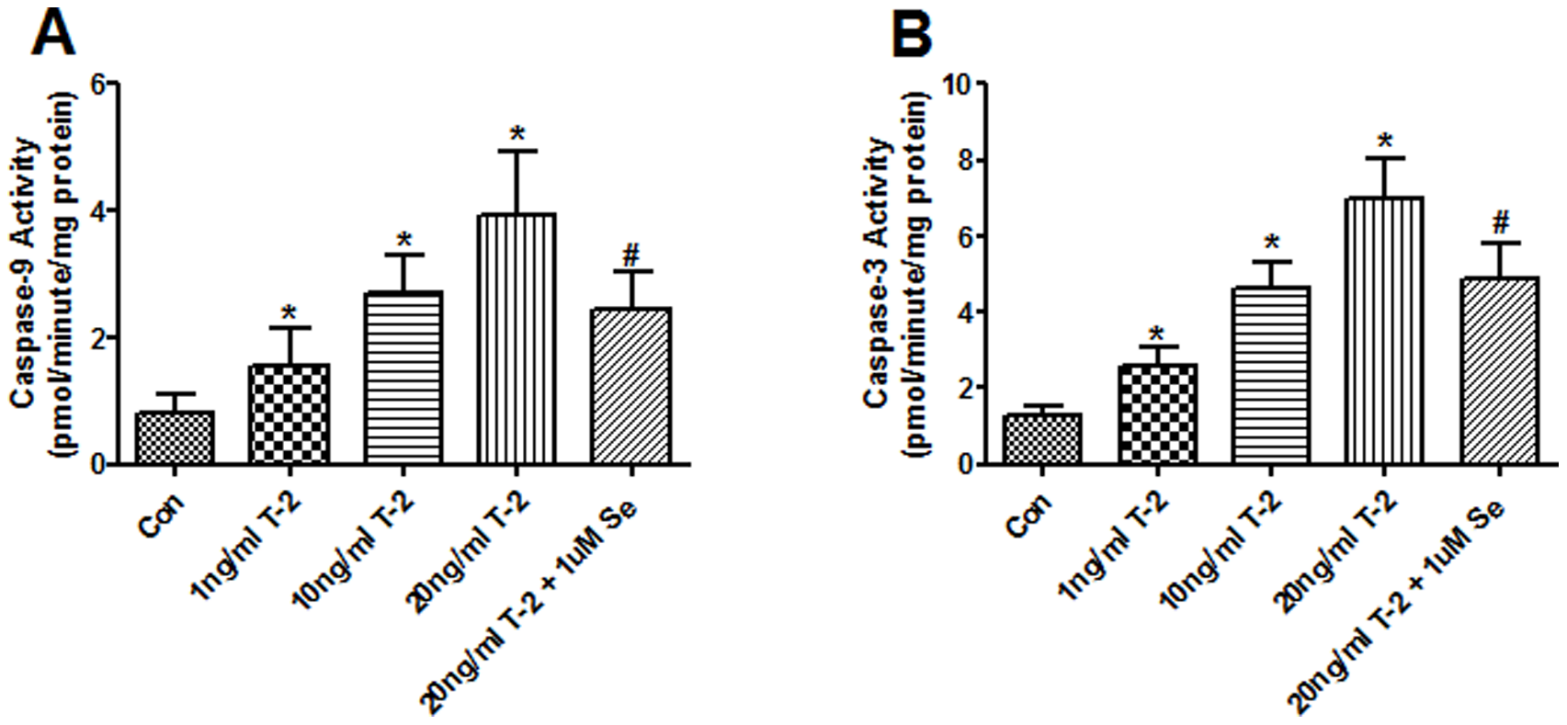
The effects of T-2 toxin on caspase-9 and caspase-3 activities in human chondrocytes. Cells were treated with 1, 10, 20 ng/ml T-2 toxin and 1 uM Na_2_SeO_3_+20 ng/ml T-2 toxin for 5 d. The activities of caspase-9 (**A**) and caspase-3 (**B**) were measured using the substrate Ac-LEHD-pNA and Ac-DEVD-pNA, separately. *P<0.05 vs. control; #*P*<0.01 vs. 20 ng/ml T-2 toxin group.

Cellular cytochrome c release and specific caspase-9 and 3 activations were analyzed to evaluate if mitochondria-related apoptotic pathway are involved in chondrocytes treated by T-2 toxin. The release of cytochrome c from mitochondria to the cytosolic was significantly increased in10 and 20 ng/ml groups (Fig. 4A and B). However, cytochrome c expression in cytosolic was significantly decreased in the group pretreated with Na_2_SeO_3_ *vs* 20 ng/ml T-2 toxin group. As Figure 5 shows that T-2 toxin dose-dependently increased caspase-9 and 3 activities in 1, 10 and 20 ng/ml. Similar to cytochrome c, this increase could be inhibited by Na_2_SeO_3_.

**Figure 6:**
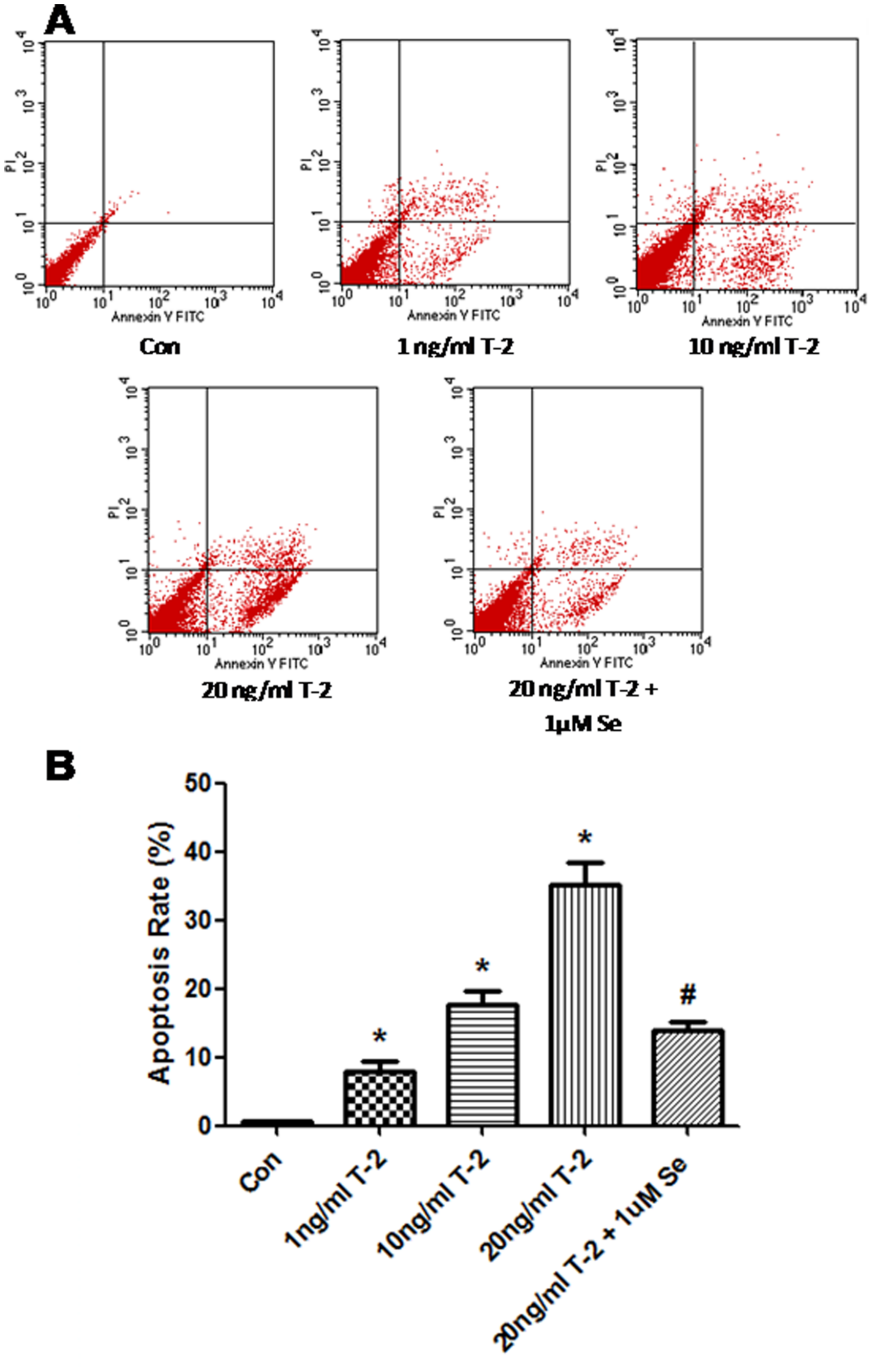
Effect of T-2 toxin on apoptosis in human chondrocytes cultured for 5 days. The apoptosis rate was determined by FCM assay. *P<0.05 vs. control; #*P*<0.01 vs. 20 ng/ml T-2 toxin group.

To explore the effects of T-2 toxin -induced human chondrocytes apoptosis, we tested apoptosis by using Annexin- V-FITC/PI method. As shown Figure 6, when exposed to T-2 toxin (1–20 ng/mL) for 5 days, the positive percentage of the annexin stained cells increase the rate of early and late stage apoptosis in chondrocytes in a dose-dependent manner, compared with untreated control (P<0.05). It was also demonstrated that selenium was able to affect apoptosis induced by T-2 toxin, and could partly block the apoptosis of chondrocytes induced by T-2 toxin.

## Discussion

It was indicated that the dysfunction of the mitochondria play an important role in KBD chondrocyte damages in our recent study, and T-2 toxin, a possible pathogenic factor of KBD, increases Bax protein production and induces chondrocyte apoptosis [19], [20], [23], [24]. The present data from analyses of cell viability, mitochondrial function and mitochondria- mediated apoptosis reveal that T-2 toxin caused insults to human chondrocytes via a mitochondrial pathway. These evidences demonstrated a series of pathological events involved in the chondrocytes exposed to T-2 toxin, including mitochondrial respiratory chain complex dysfunction, mitochondrial depolarization, ATP depletion, oxidative stress, cytochrome c release, caspase-9 and 3 activation, and apoptotic cell death.

In vitro cytotoxicity assays are useful to define basal cytotoxicity, and are also necessary to define the concentration range for further in vitro testing [25]. Our results demonstrated that T-2 toxin induced a time- and dose-dependent inhibition of cellular proliferation in chondrocytes. All concentrations of T-2 toxin used (1–20 ng/mL) significantly decreased the cellular proliferation after 4 days of exposure to T-2 toxin. The drug has few immediate toxicities at clinically effective doses, suggesting that it is a chronic pathogenic factor,which is consistent with the pathogenesis of KBD. Moreover, our previous study observed obvious chondrocyte injuries characterized by the disruption of the cytoplasm and deposition of vesicles in model cartilage after middle (1 ng/ml) or higher concentration of T-2 toxin exposure. It is noticeable that these features of chondrocyte injuries are very similar to those observed in the patients with KBD [19].

In mitochondrial oxidative phosphorylation, electron transport is coupled by the four enzyme complexes (I–IV) in the mitochondrial inner membrane, with ATP synthesis from ADP by complex V [22]. The analysis of mitochondrial MRC activity in T-2 toxin induced chondrocytes showed a definitive and significant decrease in complexes III, IV and V (13.82%, 20.83% and 20.92%, respectively). These results differ from those that reported KBD chondrocytes show a significant reduction in the activities of complexes II, III, IV and V [20]. However, examination in more detail of those results showed a quasi significant reduction in the activity of complex II (*P* = 0.055). All these data suggest that both complex II activities could be reduced in KBD chondrocytes and in normal chondrocytes stimulated with T-2 toxin, respectively. A dysfunction in complexes compromises the electron transfer pathway and ATP production. This defect could partly be compensated by increasing the anaerobic metabolism to avoid excess production of reactive oxygen molecules, since the articular chondrocytes have a particularly active in vitro rate of anaerobic glycolysis [26].

The integrity of the mitochondrial respiratory chain complex is essential for maintaining the ΔΨm. Defects in the individual complex activities induced by special complex inhibitors or RNA interference technology, causes a deterioration of the ΔΨm [27]–[29]. This study shows that T-2 toxin can decrease the ΔΨm of chondrocytes. The proton electrochemical gradient potential plays an important role in ATP synthesis and consists of two components, the transmembrane proton concentration gradient (pH) and theΔΨm. In mitochondria, the ΔΨm component significantly exceeds the pH component and is therefore regarded as the main driving force for ATP production [30]. Therefore, T-2 toxin can decrease cellular ATP levels through suppression of the ΔΨm and complex enzyme activities in chondrocytes.

ROS is one of the mitochondrial apoptotic factors. Previous experiment reported that depolarization of the ΔΨm enhances the release of apoptotic factors, including ROS and cytochrome c, from mitochondria to the cytoplasm and drives cells undergoing apoptosis [31]. Other studies revealed that different inhibitors of the MRC induced a marked increase in the levels of ROS [32], [33]. Our previous study observed that oxidative stress and mitochondrial damage play an important role in the pathogenesis of KBD [34]. This study shows that ROS were significantly augmented following T-2 toxin administration. It is noticeable that these features of chondrocyte injuries are very similar to those observed in the patients with KBD. Therefore, T-2 toxin could increase intracellular ROS through suppression of the ΔΨm and complexes activities.

Mitochondrial dysfunction is associated with mitochondrial swelling, disruption of the outer mitochondrial membrane, and the release of proapoptotic factors such as cytochrome c. Cytochrome c released from the mitochondria binds to Apaf-1 (apoptotic protease activating factor-1) and this complex activates procaspase-9. Activated caspase-9 cleaves the downstream caspase cascade such as caspase-3 [35]. This expression is also correlated with T-2 toxin treated chondrocytes. Our recent work demonstrated that T-2 toxin induced apoptosis in chondrocytes through modulation of the Bax/Bcl-2 ratio [19]. The current study demonstrated that cytochrome c was released from mitochondria to the cytosol, and caspase-9 and caspase-3 were activated in T-2 toxin treated chondrocytes. Consequently, T-2 toxin inhibited the mitochondrial function and resulted in apoptosis of human articular chondrocytes.

The effects of T-2 toxin were partially antagonized by selenium, suggesting a potential protective effect of selenium during KBD development. Selenium is an essential element for human, animals, and other species. It is found that there is a significantly lower selenium level in the grains in KBD areas when compared with none KBD areas, suggesting a close relationship between the selenium and KBD occurrence [36]. Selenium deficiency induces dysfunction of selenoproteins. Selenoproteins, including some localized in mitochondria, have been shown to exhibit a variety of biological functions, including antioxidant functions, maintaining cellular redox balance, and heavy metal detoxification, and compromise of such important proteins would lead to oxidative stress and apoptosis [37], [38]. In the present work, supplementation of the culture medium with selenium caused a large increase in GSH concentration and GPx activity. So it is likely that the T-2 toxin damages mitochondrial function, and generate oxidative stress, which in turn, lead to tissue degeneration as observed in KBD patients. On the contrary, selenium can partly blocked T-2 toxin -induced mitochondria dysfunction and chondrocytes apoptosis because of the antioxidant functions.

In summary, T-2 toxin can modulate mitochondrial functions through inhibition of the MRC complexes activity, ΔΨm, and cellular ATP levels. Levels of intracellular ROS are also regulated by T-2 toxin. However, Selenium could reduce ROS, modify mitochondrial function and improve cell survival. These data suggest that the effect of T-2 toxin on chondrocyte apoptosis is mediated by a mitochondrial pathway. Therefore, this study presents further data to validate our hypothesis that T-2 toxin can modulate mitochondrial functions, intracellular oxidative stress, and mitochondria-related caspase activation to induce chondrocytes apoptosis. More details of the molecular mechanism of T-2 toxin on chondrocytes will be displayed in our research work in future.
